# Mechanisms of Adiponectin Action in Fertility: An Overview from Gametogenesis to Gestation in Humans and Animal Models in Normal and Pathological Conditions

**DOI:** 10.3390/ijms20071526

**Published:** 2019-03-27

**Authors:** Alix Barbe, Alice Bongrani, Namya Mellouk, Anthony Estienne, Patrycja Kurowska, Jérémy Grandhaye, Yaelle Elfassy, Rachel Levy, Agnieszka Rak, Pascal Froment, Joëlle Dupont

**Affiliations:** 1 INRA UMR85 Physiologie de la Reproduction et des Comportements, F-37380 Nouzilly, France; alix.barbe@inra.fr (A.B.); alice.bongrani@inra.fr (A.B.); namya.mellouk@inra.fr (N.M.); anthony.estienne@inra.fr (A.E.); jeremy.grandhaye@inra.fr (J.G.); pascal.froment@inra.fr (P.F.); 2 CNRS UMR7247 Physiologie de la Reproduction et des Comportements, F-37380 Nouzilly, France; agnieszka.rak@uj.edu.pl; 3 Université François Rabelais de Tours, F-37041 Tours, France; 4 Department of Physiology and Toxicology of Reproduction, Institute of Zoology and Biomedical Research, Jagiellonian University, 31-007 Krakow, Poland; patrycja.kurowska@doctoral.uj.edu.pl; 5 Assistance Publique des Hôpitaux de Paris, Hôpital Tenon, Service de Biologie de la Reproduction, F-75020 Paris, France; yaelle.elfassy@hotmail.fr (Y.E.); rachel.levy@orange.fr (R.L.); 6 Université Pierre et Marie Curie Paris 6, F-75005 Paris, France; 7 INSERM UMRS_938, Centre de Recherche Saint-Antoine, F-75571 Paris, France

**Keywords:** fertility, adipose tissue, reproductive tract, adipokines, cell signaling

## Abstract

Adiponectin is the most abundant plasma adipokine. It mainly derives from white adipose tissue and plays a key role in the control of energy metabolism thanks to its insulin-sensitising, anti-inflammatory, and antiatherogenic properties. In vitro and in vivo evidence shows that adiponectin could also be one of the hormones controlling the interaction between energy balance and fertility in several species, including humans. Indeed, its two receptors—AdipoR1 and AdipoR2—are expressed in hypothalamic–pituitary–gonadal axis and their activation regulates Kiss, GnRH and gonadotropin expression and/or secretion. In male gonads, adiponectin modulates several functions of both somatic and germ cells, such as steroidogenesis, proliferation, apoptosis, and oxidative stress. In females, it controls steroidogenesis of ovarian granulosa and theca cells, oocyte maturation, and embryo development. Adiponectin receptors were also found in placental and endometrial cells, suggesting that this adipokine might play a crucial role in embryo implantation, trophoblast invasion and foetal growth. The aim of this review is to characterise adiponectin expression and its mechanism of action in male and female reproductive tract. Further, since features of metabolic syndrome are associated with some reproductive diseases, such as polycystic ovary syndrome, gestational diabetes mellitus, preeclampsia, endometriosis, foetal growth restriction and ovarian and endometrial cancers, evidence regarding the emerging role of adiponectin in these disorders is also discussed.

## 1. Introduction

It is well known that white adipose tissue is no longer the main storage compartment of triglycerides but it is a real endocrine organ releasing a number of biologically active proteins, also known as adipokines [1]. Adipokines are considered as main regulators of the whole body energy homeostasis. One of these adipokines, named adiponectin, is recognised to play a major role in regulation of the insulin sensitivity and the pathogenesis of the metabolic syndrome. In recent years, its role in the modulation of reproductive functions has become increasingly important. There has therefore been a spate of research investigating its role in the hypothalamic–pituitary–gonadal axis but also in placenta and uterus. In this review, we will discuss the structure of adiponectin and its physiological role in the male and female reproductive tract, with a predominant emphasis on its role in several human reproductive diseases including polycystic ovary syndrome, gestational diabetes mellitus, foetal growth restriction, ovarian and endometrial cancer, endometriosis and preeclampsia.

## 2. Structure and Mechanism of Adiponectin Action

### 2.1. Structure of Adiponectin Gene and Proteins

#### 2.1.1. Adiponectin Gene

**Figure 1:**
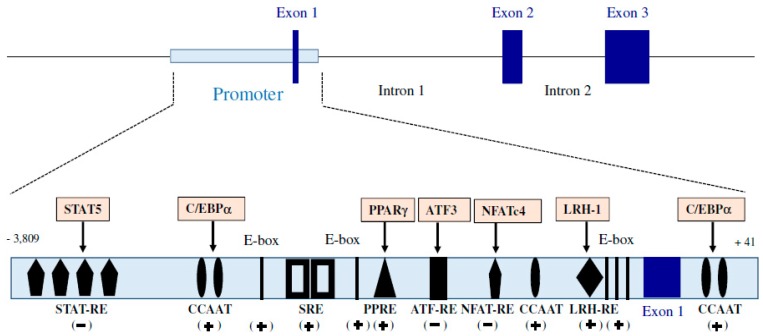
Structure of adiponectin gene and its promoter region. Binding sites (cis-elements) are STAT-RE: Signal Transducers and Activators of Transcription Response Element; CCAAT: CCAAT box is a distinct pattern of nucleotides with GGCCAATCT consensus sequence; SRE: Serum Response Element; PPRE: Peroxisome Proliferator Response Element; ATF-RE: Activating Transcription Factor-Response Element; LRH-RE: Liver Receptor Homolog 1 Response Element; E-box: Enhancer-box; NFAT-RE: Nuclear Factor of Activated T cells Response Element. Transcription factors (trans-elements) are STAT5: Signal transducer and activator of transcription 5; C.EBPα: CCAAT/enhancer-binding protein alpha; PPAR: Peroxisome Proliferator-activated Receptor gamma; ATF3: Activating Transcription Factor 3; NFATc4: Nuclear Factor of Activated T cells 4; LRH-1: Liver Receptor Homolog-1. The stimulatory (+) and inhibitory (−) roles of each transcription factor in the adiponectin gene expression are shown below the binding sites.

Adiponectin, also known as ACRP30 (adipocyte complement-related protein of 30 kDa), GBP28 (Gelatin-binding protein 28), ADIPOQ (Adiponectin, C1Q And Collagen Domain Containing) and apM1 (Adipose most abundant gene transcript 1), has been discovered as a factor produced by the white adipose tissue almost simultaneously by four different teams using different approaches. The term “adiponectin” appears in 1999 following the alignment of nucleotide sequences of these four factors [2]. In human, apM1 is a 16 kb gene consisting of three exons and two introns (Figure 1), showing sequence homologies with the genes encoding collagen VIII, collagen X and the C1q factor of complement [3]. Several regulatory regions of apM1 gene expression have been identified in one region promoter of the gene surrounding exon 1. Unlike many genes, the promoter of apM1 does not include a TATA sequence, but contains several elements of response to many transcriptional factors [4], as described in Figure 1. So, the transcriptional activity of the adiponectin gene can be regulated by many mechanisms.

#### 2.1.2. Adiponectin Protein

**Figure 2:**
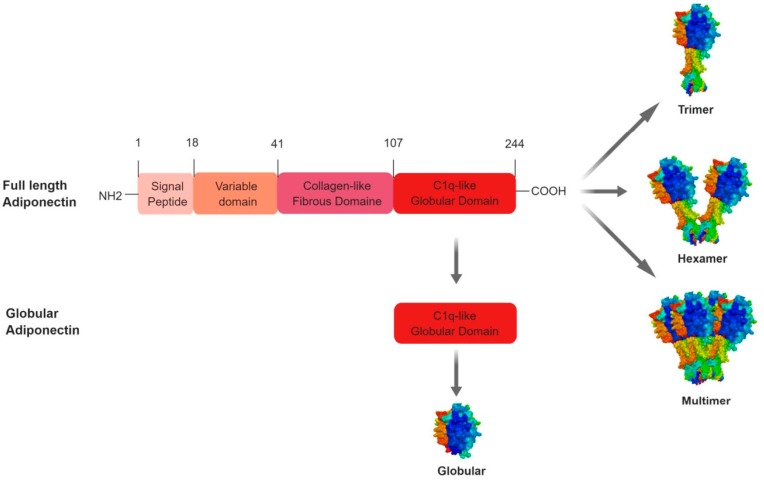
Structure and specific forms of human adiponectin (trimer, hexamer, multimer and globular).

The full length of human adiponectin (244 amino acids, 30 kDa) consists of four domains: an amino-terminal signal peptide made up of 18 amino acids, a species specific hypervariable domain of 23 amino acids, a 66-amino acid collagen-like domain consisting of 22 repeats of the motif (Glycine-X-Y) where X and Y are variable amino acids, and a 137-amino acid carboxy-terminal globular domain [5] (Figure 2). It represents the long form of adiponectin. However, it exists also a short form of adiponectin resulting from the cleavage made by an elastase secreted by monocytes and neutrophils. Several proteolytic sites have been described located within the variable sequence and the collagen domain. The short form of adiponectin preserves its globular domain integrity and can exert its effects by binding to its receptor [6] (Figure 2). In contrast to humans, mouse adiponectin is a 247-amino acid protein [7].

Adiponectin is secreted from adipocytes into the bloodstream as three oligomeric complexes including trimer (67 kDa), hexamer (complex of two trimers, 130 kDa) and a high molecular weight (300 kDa) [8] Figure 2. Adiponectin as a monomer is undetectable in native conditions. Polymerisation is therefore an essential mechanism in regulating the biological activity of the protein [9] Thus, adiponectin forms trimers (low molecular weight form or LMW) following the establishment of hydrophobic bonds between the globular domains and noncovalent interactions within α-helices of the collagenous domains [10]. The short form of the protein does not polymerise further [11]. In contrast, in its long form adiponectin trimers form hexamers (intermediate or medium molecular weight form or MMW) and much more complex structures composed of 18 or more monomers (high molecular weight form or HMW) [12,13]. This polymerisation of adiponectin requires post-translational modifications. Indeed, the formation of hexamers is achieved by the establishment of disulphide bridges between two cysteines located in the variable region of adiponectin. Experimental evidence suggests that different forms of adiponectin fractions exhibit different biological activities. For example, non-HMW adiponectin (i.e., complexes with lower molecular weight) shows stronger anti-inflammatory actions, whereas the HMW form, whose active form constitutes nearly 70% of circulating adiponectin in healthy people, may be related to insulin sensitivity [14,15].

Adiponectin is considered as the adipokine most widely present in the bloodstream. It circulates at relatively high levels (3 to 30 μg/mL) representing thus 0.01% of the total plasma proteins [16] in different species like human, pigs, dairy cows, rats, chicken and turkeys [17,18,19,20,21,22,23]. In human, MMW and HMW forms represent 90% of the protein in the circulation and the LMW form represents only 10% [24]. The globular form remains extremely minor [12]. In cows, it is well known that the plasma concentration of adiponectin reaches its minimum before calving and its maximum during early lactation [25,26,27,28,29]. Unlike rodents and humans, the main circulating form is HMW in cows [26], while trimeric forms and globular forms are not detected [28,30]. In various species, the plasma adiponectin level is likely related to reproductive pathologies (polycystic ovary syndrome, gestational diabetes mellitus, preeclampsia, endometriosis, foetal growth restriction and ovarian and endometrial cancer) that are detailed below (Section 9 of this review). Expression of the adiponectin in the body is closely related to many physiological and physiopathological processes.

Adiponectin plasma concentrations are correlated with the adipose tissue level. They are also regulated by the nutritional status. Indeed, they are increased during fasting and decreased after refeeding in rodents and sheep [31,32]. Moreover, they are higher in females compared to males in humans and rodents [21]. Adiponectin levels are lower in women under certain conditions. Indeed, Cnop et al. (2003) showed that adiponectin levels in the postmenopausal women are higher than in the premenopausal women [33], while data from Nishizawa et al. (2002) found no significant differences [21]. In mice, plasma adiponectin levels are 4-fold higher in mature female than in immature female [34]. In obese compared to control patients, adiponectin concentrations in adipose tissue and in the circulation have consistently been found to be abnormally low [35], suggesting that adiponectin is strongly associated with obesity and is a potentially important hormone in the link between obesity and women’s pathology.

#### 2.1.3. Regulation of Adiponectin Expression

Adiponectin expression can be regulated by various factors and physiological processes. As shown in Figure 1, human adiponectin gene contains binding sites for many transcription factors including PPAR (peroxisome proliferator-activated receptor gamma [36] and its coactivator PPARγ, coactivator 1α (PGC1α) [37], C/EBPα (CCAAT/enhancer-binding protein alpha) [38], LRH-1 (liver receptor homolog-1) [36], FoxO1 (forkhead box O1) [39], SREBP-1c (sterol-regulatory element-binding protein 1c) [40], ATF3 (Activating Transcription Factor 3) [41], NFATc4 (nuclear factor of activated T cells 4) [41], Id3 (inhibitor of differentiation 3) [42], STAT5 (Signal transducer and activator of transcription 5) [43] and the clock helix–loop–helix transcription factors CLOCK and BMAL1 [44]. The activation and the repression of these transcription factors are finely regulated by endogenous and exogenous signals inducing the activation of many signalling pathways in the secretory cell. Once released in the bloodstream, adiponectin exerts its physiological effects by binding to specific membrane receptors.

### 2.2. Adiponectin Receptors and Adiponectin Signalling Pathways

#### 2.2.1. AdipoR1 and AdipoR2

**Figure 3:**
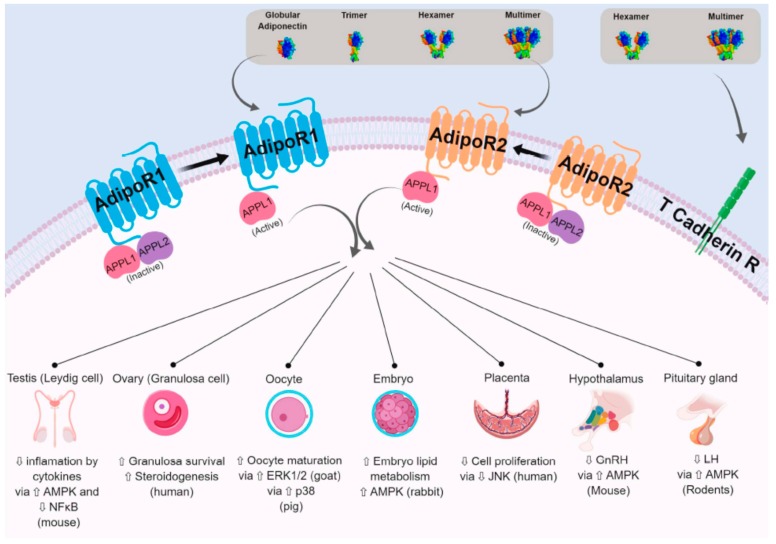
Adiponectin receptors and some examples of biological effects of adiponectin in reproductive tissues or cells. Adiponectin interacts with adiponectin receptors (mainly AdipoR1 and AdipoR2) to activate or inhibit a number of signalling pathways. T Cadherin receptor (T Cadherin R) binds the hexameric and high molecular weight isoforms of adiponectin but it has no intracellular domain. AdipoR1- and R2-dependent signalling is mediated through APPL 1 and APPL2. In the absence of adiponectin signal, APPL2 can bind to the N-terminal domain of the adiponectin receptors or it can form an APPL1/APPL2 heterodimer which prevents the APPL1/adiponectin receptors binding. On the other hand, the binding of adiponectin to its receptors favours the dissociation of this heterodimer. In peripheral tissues, adiponectin receptors have differing affinities for specific forms of adiponectin. In the reproductive tissues the affinities for specific forms of adiponectin is unknown. However, in these tissues, adiponectin regulates different biological effects through various signalling pathways. ⇧ Increase/stimulation. ⇩ Decrease/inhibition.

Adiponectin acts mainly through two seven-transmembrane domain receptors—AdipoR1 and AdipoR2—that differ from other G protein-coupled receptors. Indeed, their topology is opposite of that of the G protein-coupled receptors; their C-terminal end is located extracellularly whereas the N-terminal end is located intracellularly (Figure 3). AdipoR1 and AdipoR2 have a zinc binding motif that appears to be essential for signal transduction in the intracellular compartment [45]. They are structurally conserved (67% amino acid identity) [22]. AdipoR1 is expressed in all tissues and the highest expression is in skeletal muscles, while AdipoR2 is expressed mainly in the white adipose tissue and liver. These receptors have differing affinities for specific forms of adiponectin. AdipoR1 is a high-affinity receptor for the globular adiponectin form, and acts as a low-affinity receptor for the long form of adiponectin in skeletal muscle. In contrast, AdipoR2 is an intermediate-affinity receptor for both globular and full length adiponectin form in the liver [19,22,46].

#### 2.2.2. The Other Adiponectin Receptors

The T-cadherin receptor protein has been identified as a receptor for the MMW and HMW forms of adiponectin [47]. This membrane receptor does not have an intracellular domain. Thus, T-cadherin could regulate the bioavailability of adiponectin, rather than exerting its own effects [22]. Indeed, mice deficient in T-cadherin have increased circulating adiponectin levels, especially of the HMW form [48]. Some data also suggest that there are other AdipoR isoforms still unknown to date. AdipoR-independent effects of adiponectin have been observed in hypothalamic cells expressing the AdipoR1 and AdipoR2 receptors. Similarly, macrophages whose expression of AdipoR1, AdipoR2 and T-cadherin have been invalidated by interfering RNA still show biological effects of adiponectin [49].

#### 2.2.3. APPL1 and APPL2

Adiponectin induces activation of many signalling pathways. However, adiponectin receptors do not appear to exhibit kinase or phosphorylation domains. Indeed the targeted mutagenesis of tyrosine residues of these receptors does not induce disruption of adiponectin signalling [50]. Thus, the activation of the transduction pathways following the binding of adiponectin to its receptor involves intermediate molecules binding to adiponectin receptors in response to their conformational change. The protein APPL1 (Adaptor protein, phosphotyrosine interacting with PH (Pleckstrin Homology) domain and leucine zipper 1) has thus been identified as an adapter protein capable of binding to the intracellular domains of AdipoR1 and AdipoR2 receptors [50] (Figure 3). The binding of the APPL1 protein to adiponectin receptors is regulated by a second adapter protein, the APPL2 protein (Figure 3). In the absence of adiponectin signal, APPL2 can bind to the N-terminal domain of the adiponectin receptors or it can form an APPL1/APPL2 heterodimer which prevents the APPL1/adiponectin receptors binding [22]. On the other hand, the binding of adiponectin to its receptors favours the dissociation of this heterodimer. Thus the APPL proteins regulate the adiponectin signal according to the Yin and Yang model proposed by Wang et al. [49,51].

#### 2.2.4. Signalling Pathways Regulated by Adiponectin

Upon binding to its receptors, adiponectin activates different signalling pathways in various cell types: mitogen-activated protein kinase (MAPK), such as p38 and extracellular signal-regulated kinases 1/2 (ERK1/2); serine/threonine protein kinase (Akt); and AMP-activated protein kinase (AMPK). It is also able to phosphorylate the transcription factor, peroxisome proliferator-activated receptor alpha (PPARα). Thus, adiponectin regulates through these signalling pathways different functions in the organism [19,22]. Figure 3 shows some examples of cell signalling pathways regulated in reproductive tissues or cells.

## 3. Expression, Regulation and Effect of Adiponectin and Adiponectin Receptors in the Hypothalamic–Pituitary Axis

The hypothalamic–pituitary–gonadal (HPG) axis plays a critical role in regulating reproductive function. Gonadotropin-releasing hormone (GnRH), which is secreted by the hypothalamus, acts on pituitary gonadotrophs to stimulate luteinising hormone (LH) and follicle-stimulating hormone (FSH) synthesis and secretion, ultimately affecting the animal’s fertility. Adiponectin and its AdipoR1 and AdipoR2 receptors are expressed in the human hypothalamus and pituitary [52,53]. Adiponectin appears to play an important role in regulating the activity of hypothalamic–pituitary axis, because its deficiency disrupts FSH and LH secretion as well as LH surge [54]. Adiponectin mutation also causes significant reduction in GnRH immunoreactive neurons, which helps explain the disrupted estrous cyclicity and ovarian functions [54].

### 3.1. Adiponectin and Hypothalamus: A Role in the Fertility Regulation?

Adiponectin receptors expression in the hypothalamus has been observed in many species, including humans, rodents and pigs [52,55,56]. Adiponectin is also present in the human, mice and rat cerebrospinal fluid (CSF), suggesting an autocrine or paracrine action of this adipokine on the hypothalamic–pituitary axis [52,57,58]. In the CSF, the adiponectin trimer is the predominate form [57]. In addition, studies in mice show that peripheral intravenous application of adiponectin leads to a concurrent rise in CSF adiponectin [59]. Therefore, adiponectin does cross the blood–brain barrier, although concentrations in the CSF are approximately 1000-fold lower than that in serum [57]. Cerebrospinal fluid concentrations of adiponectin are increased during fasting and decreased after refeeding in rodent and sheep [31,32].

In the hypothalamus, GnRH neurons are key components of the reproductive axis, controlling the synthesis and release of gonadotropins. In vitro studies have notably described an inhibitory effect of adiponectin on the secretion of GnRH by hypothalamic cells through activation of AMPK [60]. Indeed, in GT1-7 cells (subset strains of GT1 cell lines) adiponectin inhibits GnRH secretion but also suppresses *KISS1* mRNA transcription [61,62]. Kisspeptins are hypothalamic neuropeptides discovered in the 2000s. The binding of kisspeptins to their KISS1-R receptors appears to be the mechanism that triggers puberty by inducing secretion of GnRH.

Thus, adiponectin appears to decrease the secretion of GnRH via the reduction of the signal emitted by kisspeptins. A more recent study showed that AdipoR2 was expressed in mouse GnRH neurons and adiponectin rapidly decreased GnRH neuronal activity in a subpopulation of GnRH neurons via a PKCζ/LKB1/AMPK signalling cascade [63].

### 3.2. Adiponectin and Pituitary: A Role in the Fertility Regulation?

Adiponectin and its receptors were also described in the pituitary of various species including human, mouse, rat, chicken and pig [53,64,65,66]. In human, adiponectin was present mainly in growth hormone (GH)-, follicle-stimulating hormone (FSH)-, luteinising hormone (LH)- and thyroid-stimulating hormone (TSH)-producing cells, whereas adiponectin receptors were located in the gonadotrophs, somatotrophs and thyrotrophs, but not in corticotrophs or lactotrophs. [53]. In cultured rat and mouse pituitary cells, adiponectin inhibited basal and GnRH-induced LH secretion [64,67]. Furthermore, it decreased the expression of the gene encoding the GnRH receptor (GnRH-R) [64]. In the porcine primary pituitary cells, adiponectin increased basal FSH release [66]. In this latter study, adiponectin also modulated GnRH and insulin-induced LH and FSH secretion dependently on the stage of the oestrous cycle. At the opposite, Sarmento-Cabral A et al., 2017 showed recently that adiponectin did not affect LH and FSH release by primary pituitary cell cultures from two normal nonhuman-primate species [68].

The presence of adiponectin receptors in the GnRH neurons and pituitary cells, and its influence on the GnRH, LH and FSH release suggests an important role of adiponectin at the hypothalamic–pituitary axis in the control of fertility in both male and female. Both LH and FSH ultimately control gonadal function. In female, ovarian follicles are stimulated by FSH to grow and mature; LH stimulates ovulation and corpus luteum formation. In men, FSH initiates, and in conjunction with high intratesticular testosterone, sustains spermatogenesis, whereas LH controls androgen synthesis by the testicular Leydig cells. As described below, adiponectin system is expressed and regulates gonadal functions.

## 4. Expression, Regulation and Effect of Adiponectin in Gonads

### 4.1. Expression, Regulation and Effect of Adiponectin System in Ovary

The involvement of adiponectin in ovary of multiple species has already been well reviewed by our and other groups [69,70]. Here we briefly summarise the major published works with an emphasis on differences between human, rodents and agronomic species.

#### 4.1.1. Plasma and Follicular Fluids Profiles

Adiponectin is detected in follicular fluids (FF). Adiponectin levels are higher in FF than in plasma in women [71], and the opposite is observed in cows [30]. In human, FF adiponectin concentrations are positively correlated with the serum values [71,72,73]. In addition to the differences in adiponectin concentrations, adiponectin isoform distribution varies between the serum and FF compartments in women. Indeed, the HMW fraction is significantly higher in serum than in FF [72]. Moreover, adiponectin levels are lower in FF from women with repeated implantation failures [74]. Taken together, all these data suggest that ovarian cells could produce adiponectin and FF adiponectin could be involved in the success of the techniques of medical assistance to procreation.

#### 4.1.2. Expression in Ovarian Cells

In granulosa cells, adiponectin expression is low and almost undetectable in humans, rodents and chickens [17,69]. However, adiponectin is strongly produced by human theca cells and even more so during follicular maturation. In contrast, the expression of AdipoR1 and AdipoR2 is greater in granulosa cells than in theca cells of hens [17]. In addition, adiponectin, AdipoR1 and AdipoR2 are present in the corpus luteum of mammalian species (human, rat, cow and sows) [69]. In avian species, the expression of adiponectin in granulosa cells is positively correlated with the weight of F3 preovulatory follicle and is upregulated in ovarian tissues during the laying period compared with the prelaying period [75,76].

#### 4.1.3. Regulation by Physiologic Status

As adiponectin system was simultaneously involved in metabolism and reproduction, modulation of the body energy status may regulate their expression in ovarian tissues. The expression pattern of adiponectin and its receptors increases in bovine granulosa cell during follicular development and the opposite was observed in bovine theca cells [77]. In human, stable levels of plasma adiponectin have been observed during the phases of the physiological menstrual cycle [78], whereas Galván and coworkers have shown lower plasma adiponectin levels in the luteal than in the follicular and the mid-cycle phase [79]. So, the plasma adiponectin profile is contradictory during the menstrual cycle in women. In pig, both gene and protein expression of adiponectin are enhanced during the luteal phase of the cycle [20]. More recently, we demonstrated that the plasma adiponectin concentration is higher in cows fed high energy diets than cows fed low energy diets presenting reproductive defects [29]. In sheep, feeding restriction increases circulating level of adiponectin and the expression level of both AdipoR1 and AdipoR2 in ovary [32]. On the other hand, the expression of AdipoR1 and AdipoR2 is decreased in theca cells of hens fed with fish oil supplementation, while the expression of AdipoR2 is increased in restricted hens [76]. Thus, adiponectin system is modulated by the energy status in various species.

#### 4.1.4. Regulation by Hormones

Hormones may regulate the production and action of adiponectin at different levels: adiponectin secretion, adiponectin receptor expression and cellular responses. In humans, LH treatment increases the level of adiponectin FF as well as in theca and granulosa cells [20,69,80,81]. In addition, FSH and hCG (a substitute for LH) treatment contribute to activate LH receptors and consequently upregulate by more than 2-fold the expression of AdipoR2 (but not AdipoR1) in human granulosa cells [69]. Conversely, an hCG injection increases the expression of adiponectin and AdipoR1 (but not AdipoR2) genes in rat ovaries [17,69]. Furthermore, the expression of AdipoR2 is increased by LH and reduced by IGF1 in bovine theca cells [69,82].

#### 4.1.5. Effect on Steroidogenesis

Adiponectin can modulate and mediate the actions of hormones production by ovarian cells. In mammals, numerous studies have shown beneficial effects of adiponectin on various physiological functions. The work published by our team has clearly demonstrated an effect of adiponectin on the steroidogenesis of ovarian cells trough variability across species. In human granulosa cells, adiponectin enhances the secretion of progesterone and oestradiol in the presence of FSH or IGF-1 [71,83]. Furthermore, depletion of adiponectin gene in mice disturbs steroidogenesis, follicular development and reduces fertility [54]. In cattle, adiponectin inhibits insulin-induced steroidogenesis in granulosa and theca cells [82,84]. In hens, adiponectin increases IGF-1-induced progesterone production by granulosa cells from F2 and F3/4 follicles and decreases LH or FSH-induced production by granulosa cells from F3/4 follicles [17].

Adiponectin also inhibits synthesis androgens including androstenedione in the murine ovary [85], as described in Section 9.1.1. In women with polycystic ovary syndrome, characterised by hyperandrogenism, circulating levels of adiponectin are decreased [86]. Adiponectin and its receptors are also present in the male reproductive tract.

### 4.2. Expression, Regulation and Effect of Adiponectin System in Testis

#### 4.2.1. Blood Plasma and Seminal Fluid Profiles

Seminal fluid (SF) is the male body fluid related to reproduction. It contains adiponectin at concentrations approximately 66- and 180-fold lower than serum in men and bulls, respectively [30,87]. In addition, a positive correlation between the adiponectin concentrations in both SF and blood plasma was observed suggesting that adiponectin is transferred from the blood to testis tissue, particularly via gaps in the blood–testis barrier.

#### 4.2.2. Expression in Testicular Cells

Adiponectin and adiponectin receptors are expressed in human testes and more precisely in the Leydig cells. Adiponectin receptors are also present in the spermatozoa [88,89,90]. AdipoR2 null mice demonstrated atrophic seminiferous tubules with aspermia (lack of semen) and enlarged brains, but displayed normal testosterone levels; whether these testicular defects reflect central or peripheral responses to the loss of AdipoR2 signalling remains unknown [91]. Expression of adiponectin and its receptors (AdipoR1 and AdipoR2) declines significantly in the testis of old mice [92].

Thus, an adequate concentration of adiponectin and its receptors may be required for normal testicular functions and adiponectin treatment could be a promising antiageing therapy promoting normal reproductive activities in the testis of aging mice.

#### 4.2.3. Regulation by Physiologic Status

In chicken, the expression of AdipoR1 and AdipoR2 mRNA is modified during the puberty; the expression of these two mRNA is increased in adulthood compared to prepubertal animals [93]. This suggests that the sexual maturation induces an upregulation of testicular adiponectin receptors genes expressions. AdipoR2 protein expression is also increased in Leydig cells during the puberty in rats [89,94] and mouse making the cells more sensitive to circulating adiponectin. Moreover, in the mouse, it has been shown that the serum concentration of adiponectin is also increased during this period [24,95].

#### 4.2.4. Regulation by Hormones

Several studies have shown a link between the steroid secretion and adiponectin. For example, an ablation of gonads in adult male mice led to an increase of circulating adiponectin [95,96]. However, when an injection of testosterone was performed on the same animals, the levels of circulating adiponectin were restored [96]. In men with hypogonadism, high concentrations of serum adiponectin were reduced by androgen supplementation [97]. A study in the rat has shown a relationship between testosterone and adiponectin. In this study, a developmental exposure to isoflavones has increased serum adiponectin levels and decreased serum testosterone levels [94]. The testis extract from the pig, enhanced adiponectin secretion in adipocyte through the peroxisome proliferator-activated receptor signalling pathway [98]. Taken together, these studies suggest that a reciprocal relationship and a possible regulation exist between gonadal steroid hormones and adipose tissue-derived factors.

#### 4.2.5. Effect on Steroidogenesis, Lactate Production and Cytokine-Mediated Cytotoxicity

Adiponectin regulates both spermatogenesis and steroidogenesis in adult testis via its two receptors, AdipoR1 and AdipoR2 [89,99]. Indeed, in vitro experiments showed that adiponectin acted directly in Leydig cells to decrease androgen secretion, which was associated with inhibition of the StAR protein in Leydig cells [94].

Following adiponectin binding, AdipoR1 and AdipoR2 activate downstream targets such as AMPK, PPAR-α, and MAPK [19]. In the testis, AMPK and PPAR-α signalling pathways have been shown to be functional and involved in the regulation of steroidogenesis [100]. Therefore, adiponectin could interact through these signalling pathways to alter testosterone production.

However, adiponectin did not modulate anti-Mullerian hormone (AMH) transcript levels [101]. Another important role of adiponectin is to maintain insulin sensitivity by stimulating glucose uptake in the testes [99]. Indeed, intratesticular glucose level was shown to be associated with testicular functions like testosterone production [102]. Furthermore, adiponectin administration ameliorates testicular mass and functions in aged mice by enhanced expression of insulin receptor, antioxidative enzyme activity, testosterone synthesis and glucose and lactate uptake by enhanced expression of transporters GLUT8 (glucose transporter) and MCT2 & MCT4 (lactate transporters) [92].

As potent anti-inflammatory mediators, adiponectin has been demonstrated to protect Leydig cells against cytokine-mediated cytotoxicity, acting as a testicular defence mechanism to attenuate the negative impact of proinflammatory molecules, particularly those released by macrophages (e.g., interleukin 1 (IL-1), tumour necrosis factor alpha (TNF-α) and interferon gamma (IFN-γ)) on steroidogenesis [103].

Thus, while adiponectin signalling appears to be present in male gonadal tissue, the extent to which this signalling contributes to normal testicular function and fertility potential need to be clarified.

## 5. Expression, Regulation and Effect of Adiponectin System in Gametogenesis (Oocyte and Spermatozoa)

### 5.1. Oocyte

**Figure 4:**
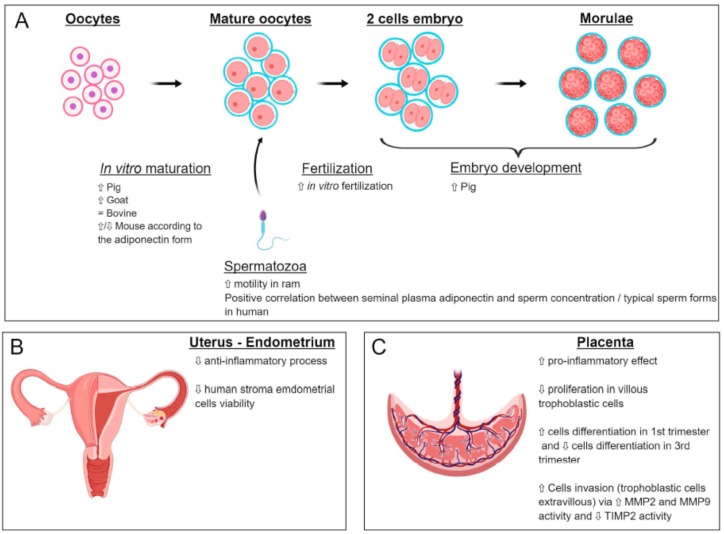
Effects of adiponectin on in vitro maturation and embryo development (**A**), uterus (**B**) and placenta (**C**). ⇧ Increase/stimulation. ⇩ Decrease/inhibition. = no effect.

The expression of adiponectin (gene and protein) was found in the oocytes of rats [17] and cows [77,84], whereas that of AdipoR1 and AdipoR2 has been shown in oocytes of cows [84], pigs [104], goats [105] and rats [17]. Several studies have shown that adiponectin supplementation during in vitro maturation (IVM) of human, mouse, goat and swine oocytes exerts positive effects on meiotic progression and initial embryonic development [104,106,107] (Figure 4A). In goat oocytes, adiponectin has a positive effect on the meiotic maturation through the classical MAPK pathway [105]. In contrast, no significant effects of adiponectin were observed on bovine IVM, cleavage and blastocyst formation rates [84] (Figure 4A). These results indicate that species differences may exist with regard to the specific oocyte response to adiponectin. In human, a decrease in DNA methylation levels in the promoter of adiponectin has been described in response to glucose IVM exposed to 10 mM glucose as compared to controls [108].

### 5.2. Spermatozoa

#### 5.2.1. Localisation of Adiponectin and Its Receptor

The presence of adiponectin receptors on spermatozoa has been reported by Kawwass et al. 2015 [70]. In bulls, adiponectin is abundantly found on flagellum whereas AdipoR1 can be observed particularly on the equatorial and acrosome regions, and AdipoR2 on the sperm head region and on equatorial line [109].

#### 5.2.2. Role of Adiponectin on Sperm Motility and Capacitation

In bull, plasma adiponectin concentration and spermatozoa mRNA abundances for AdipoR1 and AdipoR2 are positively related to sire conception rate [109]. In ram, an association between adiponectin and its receptors and sperm motility parameters has been reported [110] (Figure 4A). In human, adiponectin levels in seminal plasma have been shown to be positively correlated with sperm concentration, sperm count and percentage of typical sperm forms [87] (Figure 4A). After capacitation, the levels of adiponectin and its receptors are lowered, suggesting a direct role on sperm motility [87].

## 6. Expression, Regulation and Effect of Adiponectin System in Embryo Development and Implantation: The Evolution of the Adiponectin System during Pregnancy

### 6.1. Adiponectin System during Embryo Development

Adiponectin and its receptors are expressed in embryos at different stages of development, in different species of mammals, chickens and fishes [111,112].

Kim et al. (2011) demonstrated, in mouse, the expression of adiponectin mRNA in 2-cell and 8-cell embryos [113]. The receptors were detected at all stages of the preimplantation embryo, although levels were lowest at the blastocyst stage. AdipoR1 mRNA level was raised in 8- to 16-cell embryos. In morulas and blastocysts, the level of adipoR1 mRNA was significantly higher than in oocytes. AdipoR2 mRNA level was lower in 4-cell embryos and 8- to 16-cells embryos than in oocytes, and significantly increased in morulas and blastocysts [114]. By in situ hybridisation, adiponectin mRNA was detected in the mouse embryo at day 7 and day 8. In bovine embryo, AdipoR1 was clearly expressed but AdipoR2 and adiponectin were weakly present and undetectable, respectively [84].

The effects of adiponectin on in vitro oocyte maturation and early embryo development were assessed in different species (Figure 4A). In bovine, when culture medium of embryos was supplemented with recombinant adiponectin, any effect of adiponectin was observed in the 48 h-cleavage and day 8 blastocyst rates [84]. In mouse, when 4-cell embryos were cultured in vitro and supplemented with 10 µg/mL of different isoforms of adiponectin, most of the embryos in all groups reached the blastocyst stage; however, the full-length and the trimeric isoforms had opposite effects on the embryo distribution. With the full-length isoform, the proportion of embryos with lower cell numbers decreased while the proportion of embryos with high cell numbers increased. Opposite results were observed with the trimeric isoform [114]. In pig, when oocytes were matured in vitro in medium alone, and then cultured for 7 days with adiponectin, development to the blastocyst stage was significantly improved compared to the control group (medium alone). These results provide evidence that adiponectin has positive effects in both oocyte maturation and embryo culture in this species [104] (Figure 4A). Furthermore, a recent study shows that in pig embryos, the methylation level of AdipoR2 increased in response to female nutritional restriction [115], suggesting that the nutritional status of the mother can affect the adiponectin system in the offspring.

Mammalian preimplantation embryos contain lipid droplets [116] that serve as an energy source. They influence cell–cell interactions, cell proliferation and intracellular transport mechanisms [117]. However, excess lipid accumulation above the normal level is linked with impaired embryo quality due to cellular dysfunction and/or cell death caused by increased lipid peroxidation and mitochondrial dysfunction. In rabbit, adiponectin regulates embryonic lipid metabolism by AMPK signalling [118].

### 6.2. Evolution of Serum Adiponectin during Pregnancy

After several controversies, it is now well established that adiponectin is not a placental hormone [119,120]. Maternal adiponectinemia is constant throughout pregnancy and results mainly from adipocyte production. However, a decrease in circulating adiponectin levels is observed after delivery [121]. This suggests that placental factors contribute to increased adiponectinemia early in pregnancy and persist until parturition. It has also been shown that the HMW form of adiponectin is present in the bloodstream of the pregnant woman compared to the nonpregnant woman [122].

## 7. Expression, Regulation and Effect of Adiponectin System in Endometrium, Placenta and Relation between the Foetus and Mother

### 7.1. Expression and Effects of Adiponectin on Uterine Functions

The role of adiponectin in the endometrium is relatively unknown. The AdipoR1, AdipoR2 and adiponectin receptors themselves are present in this tissue [123,124]. A variation in AdipoR1 and AdipoR2 protein expression was measured during the menstrual cycle. Specifically, this expression is maximal in the middle of the secretory phase of the cycle, corresponding to the period of uterine receptivity to the embryo [123]. This study therefore suggests an important role of the adiponectin signal during human embryonic implantation. This hypothesis has been reinforced by a study showing that the endometrium of women with repeated implantation failures underexpresses AdipoR1 and AdipoR2 compared to fertile endometrium [124]. Adiponectin would also exert an anti-inflammatory effect in the endometrium by inhibiting the production of proinflammatory cytokines such as IL-6, IL-8 and MCP-1 (monocyte chemotactic protein-1) [123]. Finally, adiponectin decreases cell viability of human endometrial cells [125] (Figure 4B).

### 7.2. Expression and Effects of Adiponectin on the Placenta

Adiponectin appears to exert an endocrine action (via adipose tissue) or paracrine (via the endometrium) in the placenta. Thus, it regulates many placental processes:

• Inflammatory Response:

Interestingly, while its anti-inflammatory role has been described in many organs, including the endometrium, it appears that adiponectin exerts a proinflammatory effect in the third trimester placenta. Adiponectin induces the production of CD24 and Siglec-10 inflammatory molecules and interleukins IL-8 and IL-1β by trophoblasts derived from term placenta [126]. These proinflammatory effects were also observed during the in vitro culture of placental explants of the third trimester, in the presence of adiponectin. The authors observed an increase in the secretion of interleukins IL-6 and IL-1β and TNF-α via the NF-κB pathway [127]. These factors may be necessary to trigger the immunotolerance phenomenon in the mother (Figure 4C).

• Cell Proliferation

Adiponectin exerts its “classic” role of antiproliferative hormone in villous trophoblastic cells in the first trimester [128]. These results are also observed in the placenta at term, where adiponectin reduces the number of cells entering into mitosis by control of the MAPK pathway [129] (Figure 4C).

• Cell Differentiation

Adiponectin stimulates the biochemical (secretion of hCG and leptin secretion) and morphological (increased expression of syncytin-2 and decreased expression of E-cadherin) differentiation of first-trimester villous trophoblasts early (obtained before the arrival of the blood in the intervillous chamber) [120]. On the other hand, it inhibits the biochemical differentiation of villous trophoblastic cells from “late” first-trimester placentas and third-trimester placentas [119,130]. However, it has no effect on morphological differentiation in the term placenta [130] (Figure 4C).

• Cellular Invasion

Adiponectin increases the invasive abilities of trophoblastic cells by stimulating the activity of metalloproteases MMP-2 and MMP-9—two major enzymes of the invasion process—which digest the extracellular matrix of the endometrium. These enzymes thus promote the migration of trophoblastic cells within the deciduous. At the same time, adiponectin decreases the expression of the metalloprotease inhibitor, TIMP-2 [119] (Figure 4C).

### 7.3. Relation between Foetus and Mother

Recurrent spontaneous abortion (RSA) is associated with abnormal maternal tolerance to the semiallogenic foetus. A recent study shows that recombinant adiponectin therapy improves pregnancy outcome in a murine model of abortion by expanding the Treg cell population and function and decreasing the Th17 cell population and function via a p38MAPK-STAT5 pathway. This therapy reduced the abortion rate in abortion-prone model. Recombinant adiponectin administration induced the expression of AdipoR1 and AdipoR2 mRNA at the maternofetal interface [131].

Throughout the entire first trimester of pregnancy, foetal growth is sustained by endometrial secretions, i.e., histiotrophic nutrition. Endometrial stromal cells (EnSCs) accumulate and secrete a variety of nutritive molecules which are absorbed by trophoblastic cells and transmitted to the foetus. Glycogen appears to have a critical role in the early stages of foetal development, since infertile women have low endometrial glycogen levels. Duval et al., 2018 showed that adiponectin exerts a dual role at the foetal–maternal interface by promoting glycogen synthesis in the endometrium and conversely reducing trophoblastic glycogen uptake [132].

## 8. Foetus Growth

It has been observed in a mouse model that the injection of adiponectin into the mother induces a reduction in the foetal growth of young mice [133]. In humans, a negative correlation between maternal adiponectinemia and infant weight has also been observed [134]. This effect of adiponectin on foetal growth may be related to the effects of this adipokine on the expression of nutrient transporters in the placenta. Indeed, adiponectin inhibits the expression of amino acid transporters (SNAT) in the human placenta at term [135]. In the same way in the pregnant rat, adiponectin reduces the expression of the GLUT3 glucose transporter and that of lipoprotein lipase transporting fatty acids in the placenta [136]. Similar results were observed in the mouse. Indeed, chronic administration of adiponectin during pregnancy reduces placental transport of amino acids in this species [133]. Thus, by inhibiting the transport of nutrients, adiponectin appears to negatively regulate foetal growth.

It has been suggested that the hyper-growth pattern of the foetus when there is maternal obesity may be due to the relatively lesser maternal concentrations of total and HMW adiponectin. This endocrine and physiological paradigm may result in increased insulin and mammalian target of rapamycin complex 1 (mTORC1) placental signalling, as well as an upregulation of transplacental glucose and sodium-coupled neutral amino acid transporters (GLUT and SNAT) [133,137,138]. Furthermore, nutrients available for foetal growth are greater when there is maternal hypoadiponectinemia and insulin resistance [139,140].

In pregnancies where there are not symptomatic problems, increased maternal adiponectin regulates foetal growth [135]. As pregnancy progresses, the physiologic decrease in adiponectin concentrations, as well as insulin sensitivity, result in increased amounts of nutrients from the maternal to foetal circulation, increasing foetal growth [133,135,138]. With maternal obesity or gestational diabetes, total and HMW concentrations of adiponectin, however, are relatively less, even before pregnancy as compared to when the obesity condition does not exist [140]. Hypoadiponectinemia exacerbates the loss of insulin sensitivity and the increases in nutrient partitioning from the maternal to foetal circulation, resulting in larger foetuses and macrosomic babies [140,141,142]. The body weight of foetuses from adiponectin (−/−) dams was significantly greater than that of wild type dams at both embryonic day (E)14.5 and (E)18.5. In addition to nutrient supply, maternal adiponectin inhibits foetal growth by increasing IGFBP-1 expression in trophoblast cells [143].

## 9. Adiponectin and Reproductive Diseases:

Diseases associated with abnormal adiponectin levels are polycycstic ovary syndrome, ovarian and endometrial cancer, endometriosis, gestational diseases, preeclampsia and foetal growth restriction, all of which are associated with subfertility.

### 9.1. Ovarian Pathologies

#### 9.1.1. Polycystic Ovary Syndrome (PCOS)

Polycystic ovary syndrome (PCOS) is a very common endocrinopathy affecting 6 to 13% of women of reproductive age and one of the leading causes of female poor fertility [144]. Since Rotterdam Consensus Conference in 2003, its diagnosis requires the presence of at least two of the following features; oligo-/anovulation, hyperandrogenism and polycystic ovaries on ultrasound (corresponding to a follicle number per ovary ≥ 20 and/or an ovarian volume ≥ 10 mL in either ovary) [145]. PCOS is frequently associated with insulin resistance (IR), abdominal obesity [146] and an increased risk of developing type 2 diabetes since as many as 10% of women with PCOS develop diabetes by the age of 40 years [147]. Hyperandrogenism is the other main feature of the syndrome with elevated circulating androgen levels observed in 60 to 80% of PCOS patients [148]. Development of hyperandrogenism happens in part because high insulin levels and free insulin growth factor (IGF) stimulate androgens production by ovarian theca cells [149]. Furthermore, an increase in abdominal adipose tissue, stimulated by compensatory hyperinsulinemia, creates an imbalance in sex steroids with decreased sex hormone binding globulin (SHBG) levels and increased free androgens levels [149]. Although IR and hyperandrogenaemia are the essential abnormalities of the syndrome, mounting evidence supports that also genetic factors play a key role in PCOS pathogenesis [150].

**Figure 5:**
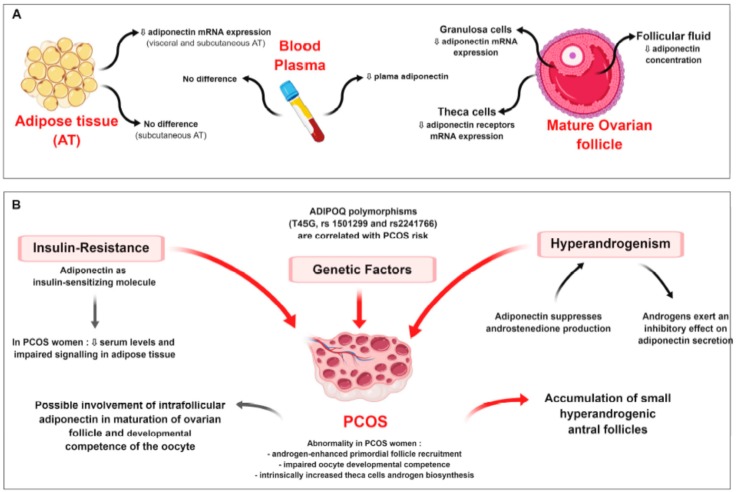
(**A**) Adiponectin system in ovary (granulosa and theca cells and follicular fluid), plasma and adipose tissue (AT) in polycystic ovary syndrome (PCOS) patient as compared to control. (**B**) Description of PCOS syndrome and possible involvement of adiponectin in this syndrome. ⇩ Decrease/inhibition.

**Figure 6:**
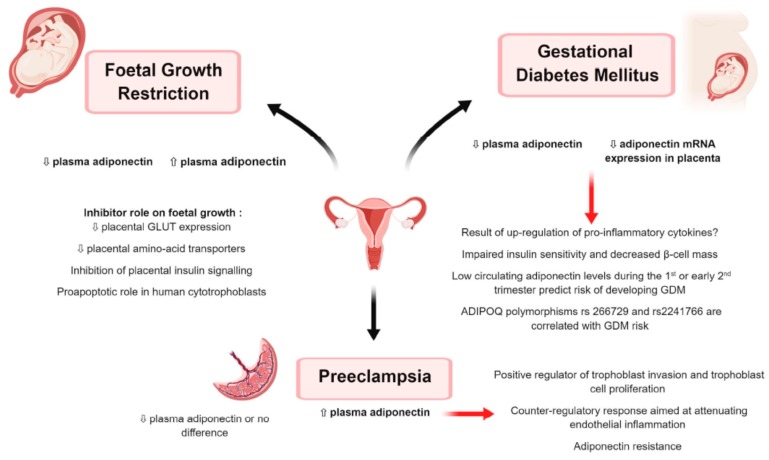
Plasma adiponectin in foetal growth restriction, preeclampsia and gestational diabetes mellitus (GDM) as compared to control patients ⇧ Increase/stimulation. ⇩ Decrease/inhibition.

The implication of adiponectin in energy metabolism as an insulin-sensitising, antiatherogenic and anti-inflammatory molecule is largely admitted. Notably, obesity and insulin-resistant states have been associated with reduced plasma adiponectin concentrations [146]. In women with PCOS, adiponectin signalling in adipose tissue seems to be impaired with decreased expression of AdipoR1 and AdipoR2, suggesting that adiponectin dysregulation may be one of the possible mechanisms responsible for lessening insulin-sensitivity [147] (Figure 6). As accumulating evidence supports a direct role of this adipokine in female reproductive tissues, altered adiponectin levels could thus be causally involved in both the reproductive and metabolic disturbances associated with PCOS (Figure 5A,B).

According to two meta-analyses [146,151], after controlling for BMI-related effects, serum adiponectin concentrations in PCOS women are lower than in non-PCOS controls. Notably, HMW adiponectin appears to be selectively reduced in women with PCOS independently of IR severity [152] (Figure 5). Nevertheless, other studies found no difference in adiponectin plasma levels between PCOS patients and controls [153,154,155,156]. Similarly, data concerning adiponectin expression in adipose tissue are controversial. Carmina et al. demonstrated that adiponectin mRNA levels were reduced in visceral and subcutaneous (SC) adipose tissue of PCOS patients compared to controls [101], while no changes of adiponectin expression in SC fat were found by Lecke et al. and Svendsen et al. [155,156] (Figure 5A).

Regarding the reproductive tissues, adiponectin concentration in follicular fluid (FF) is decreased in PCOS women [157,158,159]. In PCOS and control groups, a strong positive correlation was observed between HMW adiponectin concentrations in serum and FF samples [158]. Intrafollicular HMW adiponectin levels were 2 times lower than in plasma, suggesting a combined effect endocrine factors, including insulin and gonadotropins, rather than passive diffusion result [157]. Compared to normal ovaries, in PCOS a lower proportion of theca cells expresses adiponectin receptors [147] and granulosa cells show decreased expression of adiponectin, APPL1 [160], AdipoR1 and AdipoR2, possibly affecting follicular development and selection of a dominant follicle [158].

The downregulation of adiponectin expression in PCOS women may contribute to their characteristically lower insulin sensitivity [101] and even contribute to the hyperandrogenic environment (Figure 5B). Indeed, adiponectin suppresses production of androstenedione and key enzymes of the androgen synthesis pathway in mice ovaries [85] and cultured human theca cells [147]. Further, in granulosa cells, it increases the expression of the enzymes involved in oestradiol and progesterone synthesis [17], enhancing aromatase activity and limiting androgens production by theca cells. On the other hand, the inhibitory effect of testosterone on adiponectin synthesis has been suggested by the sexual dimorphism observed in humans, with adiponectin concentrations significantly higher in women than in men [21], and confirmed in castrated rats [161]. Similarly, in hypogonadal men, elevated adiponectin levels are reduced to rates similar to healthy individuals by a testosterone replacement therapy [97]. In vitro, androgens suppress adiponectin expression by decreasing its secretion [21], but treatment of adipose tissue with testosterone and oestradiol increases the expression of AdipoR1 and AdipoR2 [162]. According to this observation, in women with PCOS, possibly as the result of high levels of androgens, adiponectin receptors are upregulated in both subcutaneous and visceral fats, this may be a compensatory mechanism to achieve some insulin sensitivity [162] (Figure 5B).

The existence of a potentially causal relationship between adiponectin and PCOS is strengthened by genomic studies. At first, a single nucleotide polymorphism of human adiponectin precursor gene (ADIPOQ)—T45G—has been investigated in relation to PCOS, and a statistically definable correlation between the occurrence of this gene form and the ovarian disorder was found [163]. More recently, others two functional ADIPOQ polymorphisms—rs1501299 and rs2241766—were reported to be significantly correlated with PCOS risk in Caucasian women [150]. Specifically, the ADIPOQ rs2241766 TT genotype [164] and the G allele of rs1501299 [165] were associated with a significantly increased risk of developing PCOS. As previous studies have found that the presumably “protective” T allele of rs1501299 was accompanied by higher adiponectin expression, this observation further supports the hypothesis that decreased adiponectin levels are associated with PCOS [165].

Finally, data from clinical investigations in PCOS women confirm adiponectin relevant role in the physiopathology of this syndrome. Thus, Mohammadi et al. demonstrated that 8-week omega-3 fatty acid supplementation in overweight and obese PCOS patients significantly increased the mean baseline levels of adiponectin and concomitantly decreased IR [166]. This effect of omega-3 fatty acids on adiponectin has been recently confirmed by Yang et al. They also reported a significant decrease in total cholesterol, triglycerides and LDL-cholesterol, resulting in a global beneficial effect on cardiometabolic risk factors characteristic of PCOS women [167]. Further, using a dehydroepiandrosterone (DHEA)-treated PCOS mouse model, Singh et al. showed that exogenous adiponectin treatment enhanced the ovarian expression of insulin receptors and decreased theca androgen synthesis [168], which was accompanied by restored ovulation and normalised circulating androgens and glucose levels [169]. Thus, systemic adiponectin treatment could be even a promising therapeutic aid for PCOS management.

#### 9.1.2. Ovarian Cancers

Ovarian cancer is the most lethal gynaecologic malignancy among women, with an estimated 150,000 annual deaths [170]. However, due to the unspecific and inconspicuous symptoms in the early stage of ovarian cancer, there are no effective and accurate detection methods for this disease [171]. There are many types of ovarian cancer that originate from different ovarian cell types [172], including mucinous ovarian cancer, epithelial ovarian cancer, germ cell cancer, stromal cell cancer (which forms from the cells that secrete female hormones), ovarian endometrioid adenocarcinoma, clear cell carcinoma, squamous cell carcinoma and serous carcinoma [173]. Epithelial ovarian cancer, the most common ovarian malignancy, originates in the epithelial cells on the surface of the ovary and accounts for 85–89% of ovarian cancers. Germ cell cancer accounts for only 5% of ovarian cancers and originates from the cells of any one ovary. This rare cancer affects mainly adolescent girls and young women. Two other rare cancers that account for 7% of all ovarian cancers are interstitial and endocrine ovarian tumours.

Literature data have found that lower adiponectin levels are associated with higher incidence of various human cancers, such as ovarian, endometrial and breast cancers [174,175]. The inhibitory effect of adiponectin on the proliferation of several types of cancer cells has also been reported [176,177]. Brakenhielm et al. (2004) found that adiponectin inhibits primary tumour growth and is linked to decreased angiogenesis [177]. These findings suggest that adiponectin may be the link between obesity and increased cancer risk in women. The expression of AdipoR1 and AdipoR2 has been reported in a human granulosa tumour KGN cell line [83] and in various epithelial ovarian cancer cell lines. Their expression in these cell lines was lower than in the granulosa tumour cell line (COV434) [178]. Li et al. (2017) illustrated that epithelial ovarian cancer patients with AdipoR1-positive expression survived longer than those with AdipoR1-negative expression [179]. The last study of Hoffmann et al. (2018) indicated that adiponectin decreased epithelial ovarian cancer cell proliferation, and that this effect was independent of apoptosis [178]. Nagaraju et al. (2016) proposed that adiponectin action on ovarian cancer can be induced through activation of AMPK/PKA pathway and PPARγ regulation [180].

### 9.2. Uterine/Endometrial Diseases

#### 9.2.1. Endometriosis and Endometrial Cancer

Endometriosis corresponds to ectopic implantation and a high invasiveness of the endometrial tissue. Some studies have indicated that serum adiponectin level decreases in women with endometriosis [181] and endometrial cancer [182]. Also, adiponectin level in peritoneal fluid of endometriosis patients decreased dramatically in advanced endometriosis [183]. Takemura et al. (2006) compared adiponectin concentrations in serum and peritoneal fluid in women with and without endometriosis [123]. They reported that adiponectin concentrations were lower in women with endometriosis than in those without endometriosis. However, Pandey et al. (2010) observed similar adiponectin levels in women with pelvic endometriosis compared to women without endometriosis [184]. Similar results were reported by Choi et al. (2013), who did not find any difference in the expression of adiponectin or AdipoR in normal endometrium and ovarian endometrioma [185]. Adiponectin inhibit endometrial stromal cell proliferation in dose and time dependant manner, and cause cell death, suggest as antiendometriosis agent [125].

So, adiponectin could be a beneficial factor to limit the endometriosis. However, further studies are necessary to better understand its effects in this gynaecologic disease.

#### 9.2.2. Endometrial Cancer

Endometrial (uterine) cancer starts in the layer of cells that form the lining (endometrium) of the uterus. Over 80% of endometrial cancers are adenocarcinomas (endometrioid). Endometrial cancer is most commonly found in women 55 years and older and rarely occurs in women below 45 years of age [180]. Women with high leptin levels, lower circulating levels of adiponectin in serum due to obesity, hyperinsulinaemia, and high leptin/adiponectin ratio have the highest risk of developing endometrial cancer [186]. Several study documented that adiponectin and obesity act independently in promoting endometrial cancer [187,188]. High circulating levels of adiponectin are related to a reduced risk of developing endometrial cancer, independent of the other risk factors such as insulin resistance and hypothyroidism that cause obesity [189]. The effect of adiponectin and obesity, synergistically, was associated with a 6-fold increase in the risk of developing endometrial cancer. Study of Hyun-Seuk Moon et al. documented that the adiponectin receptors expression is similar in normal and cancerous tissues, but AdipoR1 was higher than that AdipoR2 in the human endometrial cancer cell lines KLE and RL95-2 [190]. Moon et al. hypothesised that adiponectin mediates activation of the AMPK pathway by LKB1 (an adapter molecule with growth-suppressing effects on tumour cells) [190]. Adiponectin-mediated AMPK activation inhibits cell proliferation, colony formation, and adhesion and invasion properties of endometrial cancer cells [191], and inhibits angiogenesis and the neovascularisation process in mouse [177]. Decreased expression of cyclin D1 and E2, different pro-growth regulators of cell cycle, and the signalling proteins ERK1/2 and Akt are all associated with PTEN (phosphatase tensin homolog, tumour suppressor gene) activity and LKB1-mediated adiponectin signalling in inhibiting endometrial carcinogenesis. These results suggest that additional studies are needed to determine the significance of adiponectin and adiponectin receptors as prognostic markers and therapeutic targets in endometrial cancer [180].

### 9.3. Gestational Pathologies

#### 9.3.1. Gestational Diabetes Mellitus (Figure 6)

Gestational diabetes mellitus (GDM) is defined as “diabetes first diagnosed in the second or third trimester of pregnancy that was not clearly overt diabetes prior to gestation” [192]. According to last International Diabetes Federation estimation, it affects approximately 14% of pregnancies worldwide, representing ←18 million births annually [193]. During pregnancy, GDM can result in serious complications for both mother and child, including preeclampsia, preterm birth, stillbirth, macrosomia and hypoglycaemia in the newborns. Moreover, although it usually resolves following delivery, in the long-term, women with a past history of GDM and babies born of GDM pregnancies are at increased risk of obesity, type 2 diabetes mellitus (T2DM) and cardiovascular diseases [193].

In healthy pregnancy, insulin sensitivity (IS) increases during early gestation to promote glucose uptake into adipose stores in preparation for the energy demands of later pregnancy. As pregnancy progresses, however, IS lessens under the effect of several local and placental hormones. As result, glycaemia is slightly elevated and glucose is readily transported across the placenta to fuel foetal growth. This physiological state of insulin resistance (IR) also promotes endogenous hepatic glucose production and lipolysis in adipose tissue, resulting in a further increase in blood glucose and free fatty acid (FFA) concentrations [193]. Pregnant women compensate for these changes through hypertrophy and hyperplasia of pancreatic β cells, as well as increased glucose-stimulated insulin secretion [194]. Failure of this compensatory response gives raise to maternal hyperglycaemia or GDM [195]. Thus, GDM is usually the result of β cell dysfunction on a background of chronic IR during pregnancy. In most cases, both β cell impairment and tissue IR exist prior to pregnancy and can progress, representing the basis for increased risk of T2DM in post-pregnancy [193]. Indeed, GDM is often considered as a prediabetic state [196].

Human pregnancy is a physiological condition characterised by decreased circulating adiponectin [197]. In late pregnancy adiponectin mRNA levels in white adipose tissue were 2.5-fold lower compared to pre pregnancy assessments, most likely suggesting reduced adiponectin production, possibly due to gestation-related adipose tissue accumulation in abdominal compartment [197]. Interestingly, lowering in total adiponectin is reflected primarily at the level of HMW adiponectin complexes resulting in decreased HMW/LMW ratio, further suggesting that HMW adiponectin is the active form of the protein [197,198]. Pregnancy-mediated adiponectin changes seem related to impairment of peripheral IS to glucose, but not to lipid metabolism as, contrary to what happens in nonpregnant women [198], in late pregnancy plasma adiponectin concentrations were independent of FFA levels under conditions of hyperinsulinemia [197].

Interestingly, during pregnancy, adiponectin is expressed and circulates in maternal and foetal compartments separately [199]. It can be found in foetal circulation at the 24th week of gestation at the earliest [200] and its levels increase in parallel with gestational age [201]. Compared to maternal plasma, umbilical vein serum adiponectin concentration was found 3-fold higher [202]. As no correlation between adiponectin levels in the maternal and foetal circulation was shown, and since there is no transplacental crossing of molecules larger than 500 Da, it is likely that different mechanisms are implicated in adiponectin production and regulation in the foetus and the mother. Notably, adiponectin may have an important role in foetal carbohydrate metabolism, especially in the presence of GDM [200]. These findings even suggest that human placenta might be an independent source of adiponectin [203]. Indeed, although the results were controversial [119], there are evidences that adiponectin and its receptors are present in rat and human placenta [136,163] and that human placenta is able to secrete adiponectin in an in vitro model [203]. Interestingly, Chen et al. demonstrated also that, compared to normal placenta, GDM placenta has significantly lower adiponectin gene expression but increased AdipoR1 levels [203].

Hypoadiponectinemia has been widely observed in women with GDM [200,204,205,206]. In particular, compared to pregnant control women, plasma adiponectin concentration in patients with GDM markedly decreased in the 3rd trimester of pregnancy, but significantly raised up 24 h postpartum [200], strongly supporting a straight correlation between hypoadiponectinemia and pregnancy-related IR. Several authors have also linked hypoadiponectinemia to the low-grade inflammatory condition typical of pregnancy further exacerbated by GDM [197,203,204], derived from white adipose tissue and placenta increased production of IL-6 and TNF-α [207]. Indeed, TNF-α is known to inhibit adiponectin synthesis ([208] and a significant negative correlation was found between this proinflammatory cytokine and adipose tissue mRNA/plasma adiponectin levels [209]. Thus, the upregulation of proinflammatory cytokines may represent an important functional link between hypoadiponectinemia and IR in GDM [197].

A recent meta-analysis showed that circulating adiponectin levels during the first or early second trimester of pregnancy were significantly lower in women who late developed GDM [210]. This result was confirmed by Illiodromiti et al., suggesting that prepregnancy and early pregnancy assessment of plasma adiponectin may improve the detection of women at high risk of developing GDM [211]. These findings also confirm that the decline in maternal adiponectin levels precedes clinical diagnosis of GDM [212], implying that women with GDM are most likely metabolically different before gestation [213]. Remarkably, the association between adiponectin levels and subsequent risk of GDM appears to be independent of adiposity in early pregnancy [212,213,214], suggesting that other pathways may be involved.

The potential role of adiponectin in pathophysiology of GDM is further supported by recent genomic studies. Indeed, the G allele of ADIPOQ gene rs266729 polymorphism is associated with an increased risk of GDM, independently of age, BMI before pregnancy and past pregnancies [215,216]. Further, the association between the G allele of rs2241766 polymorphism and GDM was independently found in a Chinese population [217], an Iranian population [218] and a Malaysian population [219]. Interestingly, the G allele of rs266729 polymorphism is associated with lower adiponectin levels and is considered a risk factor for developing T2DM [215]. Similarly, in the cohort of Han et al., women with TG or GG phenotypes presented significantly lower plasma adiponectin concentrations than TT homozygotes women [217].

Results from animal investigations strongly suggest that hypoadiponectinemia may even underlie GDM. In particular, Qiao et al. demonstrated that pregnant mice with adiponectin deficiency (Adipoq^−/−^) spontaneously developed the main characteristics of GDM, as glucose intolerance, hyperlipidaemia and foetal overgrowth [199]. Interestingly, compared to wild type, in Adipoq^−/−^ dams, despite higher blood glucose concentrations, plasma insulin levels were significantly lower as the result of decreased β cell mass. Remarkably, adiponectin reconstitution during late pregnancy restored maternal metabolism, β cell mass and foetal body weight [199]. Thus, adiponectin is most likely involved in controlling maternal metabolic adaptation to pregnancy and hypoadiponectinemia may play a causal role in the development of GDM [199]. Particularly, adiponectin may represent a factor in the expansion of β cell mass that is believed to be necessary for the maintenance of glucose homeostasis in pregnancy [195]. Indeed, adiponectin is known to enhance β cell proliferation [220] and a strong association between blood adiponectin concentration in late pregnancy and β cell function has been repeatedly found [221,222]. Moreover, ante-partum hypoadiponectinemia seem to predict postpartum IR, β cell dysfunction and fasting glycaemia, providing a means of stratifying women with GDM with respect to their future risk of T2DM [206].

In summary, adiponectin may be associated with GDM development through impaired insulin sensitivity, decreased β cell mass and attenuated anti-inflammatory capacity, thus representing a potential target for treatment or prevention of GDM [195] (Figure 6).

#### 9.3.2. Preeclampsia (Figure 6)

Preeclampsia (PE) is a severe pregnancy complication affecting 4.6% of pregnant women worldwide [223]. It is at the second or third place in the world ranking of maternal morbidity and mortality causes [224]. It is defined as the association of arterial hypertension appearing from the 20th week of gestation onward and one of the following conditions: proteinuria, maternal organs dysfunction (renal insufficiency, hepatic impairment, neurological complication or haematological disorder like thrombocytopenia or haemolysis) and uteroplacental dysfunction including foetal growth restriction [224]. The pathophysiology of PE has not yet been fully elucidated. However, the two main characteristics of the syndrome appear to be an abnormal placentation and an exaggerated maternal inflammatory response [224]. Indeed, initial incomplete trophoblast invasion and abnormal uterine spiral artery remodelling would be followed by the release into maternal circulation of placental factors, such as inflammatory cytokines and reactive oxygen species, able to trigger a broad intravascular inflammatory response resulting in endothelial dysfunction [225]. As clinical manifestations of PE regress after delivery, it is likely that placental trophoblast cells function may play a central role in its pathogenesis [226]. Like GDM, PE shares risk factors with metabolic syndrome including IR, subclinical inflammation and obesity [227], and women with a history of hypertensive pregnancy disorders present 1.4–3 times higher risk of future cardiovascular diseases compared to women with normotensive pregnancies [228]. Notably, IR was suggested to be part of the pathophysiology that links obesity and PE and would explain the increased rate of this syndrome in obese pregnant women [225].

Strong evidence supports the association between PE and hypoadiponectinemia during the first trimester of pregnancy [163]. In late pregnancy however a paradoxical significant increase in circulating adiponectin has been repeatedly found [201,229,230,231,232,233]. Indeed, this result is highly controversial, with some studies reporting hypoadiponectinemia [234,235,236] and others finding no significant difference in serum adiponectin levels during pregnancy compared to normal pregnant women [237,238]. Likewise, Haugen et al. failed to demonstrate significant differences in adipose tissue adiponectin mRNA expression in PE patients compared to healthy controls [231]. Reasons for these conflicting results include PE definition, ethnic background of patients, BMI, renal function and smoking [227]. Moreover, Takemura et al. showed that adiponectin changes between PE patients and normal pregnant women were limited to HMW isoform, since no significant difference was found in low- or medium-molecular weight isoforms [234].

Similarly, studies that evaluated the potential prediction of PE by adiponectin measurement in early pregnancy showed conflicting results [227]. Analyses of the relationship between circulating adiponectin and BMI in PE were also inconsistent. Plasma adiponectin levels decreased in women with severe PE and BMI > 25 kg/m^2^, whereas they increased in normal weight PE patients [233]. Likewise, Eleuterio et al. showed a negative correlation between serum adiponectin concentration and BMI in normal pregnant women but not in PE patients [232].

The association of genetic variations of the single-nucleotide polymorphism (SNP) type in the adiponectin gene (ADIPOQ) with IR, metabolic syndrome, GDM, T2DM and hypertension has been widely reported [239]. Interestingly, PE was found to be associated with one of the same polymorphisms, 276G>T, that correlates with ovarian disorders [163]. Notably, the TT genotype seems to be related with protection against the development of PE [240]. A significant association was also found between PE and the CT genotype of the −11377C>G polymorphism [239], suggesting that adiponectin may be involved in PE development.

Regarding the pathophysiological role of adiponectin in PE, it has been hypothesised that increased adiponectin concentrations could be part of a physiological feedback mechanism aimed at improving IS and mitigating endothelial dysfunction and cardiovascular risk associated with this syndrome [231]. Actually, adiponectin might attenuate the excessive inflammatory response in the vascular wall through inhibition of NF-κB signalling, decreased CRP (C-reactive protein) and increased nitric oxide generation [227] via endothelial nitric oxide synthase activation and superoxide inhibition in endothelial cells [241]. Further, some reports proposed adiponectin as a positive regulator in the process of trophoblast invasion by modulation of MMP/TIMP balance [232]. In particular, adiponectin showed the ability to increase MMP2 and MMP9 activity in human extra villous trophoblast cells via a reduction of TIMP2 expression [119]. Interestingly, Eleuterio et al. found a negative correlation between circulating adiponectin and MMP2 and TIMP2 in PE patients, suggesting that hyperadiponectinaemia may contribute to the systemic endothelial dysfunction characterising PE [232]. The role of adiponectin in proliferation of trophoblast cells and invasive mechanisms was also demonstrated in rat placenta, where adiponectin and ADIPOR2 expression has been repeatedly reported [136,163]. Intriguingly, some authors have also suggested that adiponectin upregulation in PE patients might represent the result of an adiponectin resistance state, as already described in different animal models [227].

In summary, until now data about the relationships between adiponectin and PE are poor. Nevertheless, its insulin-sensitising and anti-inflammatory activities and its ability in modulating trophoblast cells proliferation and trophoblast invasion led us to speculate that this adipokine might play a role in PE pathogenesis (Figure 6).

#### 9.3.3. Foetal Growth Restriction (Figure 6)

Foetal Growth Restriction (FGR) is a common complication of pregnancy affecting 3 to 9% of pregnancies in the developed world and up to 25% of pregnancies in low-middle income countries [242]. Traditionally, an estimated foetal weight less than the 10th percentile for the population at a given gestational age is highly suggestive of FGR. The main feature of this pathological condition is a placental failure to adequately supply oxygen and nutrients to the developing foetus, thus resulting in a stunted foetal growth [243] This phenomenon, named placental insufficiency, is idiopathic in up to 60% of cases and it is due to a physiological deficiency in uterine spiral arteries remodelling, resulting in restricted uteroplacental perfusion [244]. Placentas from FGR foetuses are then small and show vascular defects that seem to be associated with excessive apoptosis and impaired trophoblast invasion [245]. Moreover, the altered expression of glucose, amino acids and fatty acids carriers in placental syncytiotrophoblast contributes to reduced nutrients transport from mother to foetus [245,246,247]. Indeed, nutrient supply, a key determinant of foetal growth, depends mainly on placental nutrient transport rather than maternal nutrient levels [133]. In the foetus, hypoxia derived from placental insufficiency results in the so-called brain sparing, that is, the preferential blood flow redistribution to vital organs like brain, myocardium and adrenal glands. Prolonged foetal hypoxia reduces foetal weight and has an adverse impact on foetal organ development and vascular remodelling, resulting in increased rates of neonatal mortality and morbidity [243]. FGR is the greatest risk factor for stillbirth [248] and FGR newborns more likely present transient neonatal morbidities including hypothermia, altered glucose metabolism, polycythaemia, jaundice and sepsis [249]. Interestingly, Small-for-Gestational-Age (SGA) is also a likely risk factor for the development of metabolic complications in later life, such as obesity, high blood pressure, glucose metabolism disorders and adipose tissue dysfunction [250].

Data concerning the relationship between adiponectin concentration in maternal circulation and FGR are controversial. Some studies showed increased serum adiponectin levels [251,252], whereas others reported a negative correlation between circulating adiponectin and FGR [253,254]. Interestingly, mothers who gave birth to Large-for-Gestational-Age (LGA) children had lower plasma adiponectin levels, and hypoadiponectinemia was accompanied by a decrease in mRNA levels of adiponectin receptor AdipoR2 [255]. It is even noteworthy that, according to a very recent meta-analysis [250], blood adiponectin concentration at birth is significantly lower in SGA newborns than in healthy controls.F

Compared to adults, adiponectin concentration in human foetal circulation and umbilical cord blood is 2 to 3 times higher, suggesting that adiponectin may be involved in foetal growth. This hypothesis was confirmed by four independent studies which demonstrated that adiponectin downregulates placental nutrient transport functions. In particular, adiponectin inhibited glucose transporter GLUT1 and GLUT2 expression in human villous cytotrophoblasts [256] and downregulated GLUT3 mRNA expression in placentas of rats exposed to a chronic adiponectin treatment during pregnancy [136]. Besides, in vivo experiments showed that adiponectin chronic infusion in pregnant mice was associated with downregulation of placental amino acid transporters via inhibition of mTOR signalling and resulted in a 19% foetal weight drop [133]. The same result was found in human villous cytotrophoblasts [256]. Using a mouse model of obesity in pregnancy, Aye et al. further confirmed that maternal adiponectin supplementation prevents foetal overgrowth caused by maternal obesity by the inhibition of placental insulin and mTORC1 signalling resulting in normalisation of placental nutrient transport [138]. Surprisingly, in human villous cytotrophoblasts, adiponectin seems also to inhibit mitochondrial biogenesis and to play a proapoptotic role via caspase activity, suggesting a causative role of this adipokine in foetal growth regulation [256].

Hence, adiponectin seems to function as an endocrine link between maternal adipose tissue and foetal growth by regulating placental functions [138]. This may further explain the strong association between maternal BMI and birth weight. Indeed, based on these postulates, low adiponectin levels characterising women with obesity and GDM would remove the inhibition of this adipokine on placental insulin signalling and amino acid transport, thereby promoting increased foetal growth [133] (Figure 6).

## 10. Conclusions

Adiponectin and its receptors are largely expressed in the central and peripheral reproductive tissues in both male and female in different species. In mice, adiponectin deficiency leads to female subfertility associated to central and ovarian dysfunctions. These data suggest that adiponectin is essential for normal mouse reproduction. However, the role of the local versus systemic adiponectin in the fertility is still unclear. Moreover, the involvement of the different forms of adiponectin in reproductive tract remains also to be investigated. Interestingly, plasma and/or tissue expression of adiponectin might be associated to various reproductive diseases like PCOS syndrome, gestational diabetes, preeclampsia and uterine growth restriction. Studies on animal models and human data suggest that adiponectin could be a potential target for treatment or prevention of these pathologies. Finally, all the data suggest that additional studies are needed to determine the significance of adiponectin and adiponectin receptors as prognostic markers and therapeutic targets in different ovarian or endometrial cancers.

## Abbreviations

STAT	Signal Transducers and Activators of Transcription
*CCAAT*	*CCAAT* box is a distinct pattern of nucleotides with GGCCAATCT consensus sequence
SRE	Serum Response Element
PPRE	Peroxisome Proliferator Response Element
AP-1	Activator Protein 1
LRH-RE	Liver Receptor Homolog 1 Response Element
LMW	Low Molecular Weight
MMW	Medium Molecular Weight
HMW	High Molecular Weight
PCOS	Polycystic Ovary Syndrome
CSF	CerebroSpinal Fluid
HPG	Hypothalamic–Pituitary–Gonadal axis
AMH	Anti-Mullerian Hormone
MMP	Matrix MetalloProteinases
TIMP	Tissue Inhibitors of MetalloProteinases
PE	PreEclempsia
IGFBP-1	Insulin-like Growth Factor Binding Protein 1
hCG	human Chorionic Gonadotropin
FF	Follicular Fluid
TNF	Tumour Necrosis Factor
IFN	InterFeroN
PKA	Protein Kinase A
SF	Seminal Fluid
IVM	In Vitro Maturation
IGF-1	Insulin like Growth Factor 1
IR	Insulin Resistance
IS	Insulin Sensitivity
FFA	Free Fatty Acid
BMI	Body Mass Index
StAR	Steroid Acute Regulatory protein
T2DM	Type 2 Diabetes Mellitus
DHEA	DeHydroEpiAndrosterone
PTEN	Phosphatase and TENsin homolog
LKB1	Liver Kinase B1
NFkB	Transcription factor Nuclear Factor-kappa B
IL8/1	InterLeukin 8/1
ERK1/2	Extracellular signal-Regulated Kinases 1 & 2
MAPK	Mitogen-Activated Protein Kinases
PPAR	Peroxisome Proliferator-Activated Receptor
AMPK	AMP-activated Protein Kinase
APPL1/2	Adaptor Protein, Phosphotyrosine interacting with PH domain and Leucine zipper 1/2
BMAL1	Brain and Muscle Arnt-Like protein-1
CLOCK	Circadian Locomoter Output Cycles protein Kaput
Siglec10	Sialic acid binding ig like lectin 10
SNAT	Sodium-coupled Neutral Amino acid Transporters
mTORC1	Mammalian Target Of Rapamycin Complex 1 or mechanistic target of rapamycin complex 1
GLUT	GLUcose Transporter
MCT2/4	MonoCarboxylate Transporter (lactate transporter)
